# Extract from the Minutes of the Atlanta Academy of Medicine

**Published:** 1871-06

**Authors:** 


### *Extracts from the Minutes of the Atlanta Academy of Medicine.*

April 7, 1871.

Reports of cases being in order:

Dr. Miller reported the case of a boy æt. twelve years; had been under treatment by another physician previously; two days ago symptoms of meningitis came on; fever, pupils dilated, head drawn back and convulsious.

*Treatment*—Blister to the neck; friction to the spine with turpentine, camphor and oil; also friction to the extremities; internally, minute portions of calomel were given, resulting in catharsis. Believing fibrin was being effused on the meninges of the brain and spinal cord, carbonate ammonia was given, with the view of promoting its solution and absorption; thinks the muriate or other neutral salt of ammonia preferable for this purpose. The symptoms have improved under this treatment; now appears rational, but neck still rigid, and head thrown back; circulation better.

He had recently seen two other cases resembling meningitis. One, a young lady subject to sick-headache; was suffering from nausea and severe pain in the head, and in two hours from the first visit became insensible. The other, a lady seventy years old. The symptoms in this case indicate meningitis, but her age inclines him to doubt the existence of meningeal inflammation.

Dr. Love stated that he had found acetate and muriate of ammonia useful in such cases, and had been induced to use ammonia by a similar course of reasoning to that mentioned by Dr. Miller.

Dr. Ingraham referred to a case of cerebrospinal meningitis in a child, treated successfully by acetate of ammonia and tincture chloride of iron. He had used the same in other cases with favorable results.

Dr. Miller said he had not used iron in this disease, but thought favorably of it in anæmic subjects.

Dr. Wells stated that in the epidemic of this disease which prevailed at Mobile some years since, some of the cases treated by blisters at the onset of the affection, with decided doses of quinine, recovered; but when the treatment was delayed, generally proved fatal.

Dr. Miller stated that during the late war, he at one time supposed the cause of meningitis to be of malarial character. The results of treatment, however, disproved this theory. He then supposed it probably was the result of deficient or improper food, and a want of protection against the effects of changes in the weather. When, however, the disease was found to attack the better classes, he no longer entertained this opinion. He now desired the opinion of members as to its cause.

Dr. W. F. Westmoreland reviewed the history of influenza, in which were found cases of meningitis. He regards the disease under discussion as an aggravated form of influenza, the cause of which exists in epidemic influences. Whether this influence depends upon electrical or other peculiarities of the atmosphere, is not certainly known.

MAY 5, 1871.

Dr. Wells reported a case of phlegmasia dolens treated with hydrate of chloral. Pain in the limb was controlled by the remedy, in the dose of twenty grains every two or three hours. Carbolic acid, in the strength of ten grains to the fluid ounce of water, was used as a vaginal wash, and chlorate potassa and muriated tincture iron internally, in addition to the chloral. Being interrogated as to the difference in reliability of chloral from different manufactories, stated that the various specimens used by himself had proved equally effective.

Dr. Withers had been disappointed in obtaining the usual effects of chloral in some instances, which he attributes to the impurity of the specimen used.

Dr. Love had seen a statement in some medical journal to the effect that German manufactories produce much more reliable chloral than that made in England or America.

Dr. Logan attributed the want of activity in the American preparation to inexperience in its manufactory.

Dr. Wells thought the difference in the action of chloral from the various manufactories must be very slight, as he had uniformly obtained good results from its use.

Dr. Love desired the experience of members as to the irritating effects of chloral on the intestinal canal.

Dr. Logan had seen evidences of such action upon the alimentary mucous membrane, but was not certain that this effect is usual.

Dr. J. T. Johnson had recently used chloral successfully in palpitation of the heart, in the dose of fifteen grains.

Dr. J. G. Westmoreland desired information as to the state of dilution chloral is administered by members in the habit of using it. It being an irritant to the skin, must necessarily irritate the stomach, if not very much diluted.

Dr. Logan is in the habit of using a wineglassful of water to ten grains of the salt.

Dr. Alexander had been rather unfortunate in the use of this remedy, and advised caution in its administration. He had seen it given in the proportion of six grains to the drachm of water, when symptoms of gastritis followed.

Dr. Wells thought the effects resulting from the improper use of chloral should not deter physicians from making a proper application of the remedy.

Dr. Orme had given it in the proportion of ten grains to the ounce of water, without evidences of irritation in the alimentary mucous membrane.

Dr. Withers had used fifty grains not largely diluted, without unpleasant effects, and does not think it is likely to irritate the stomach. He is of opinion, however, that some specimens of the remedy are almost inert.

